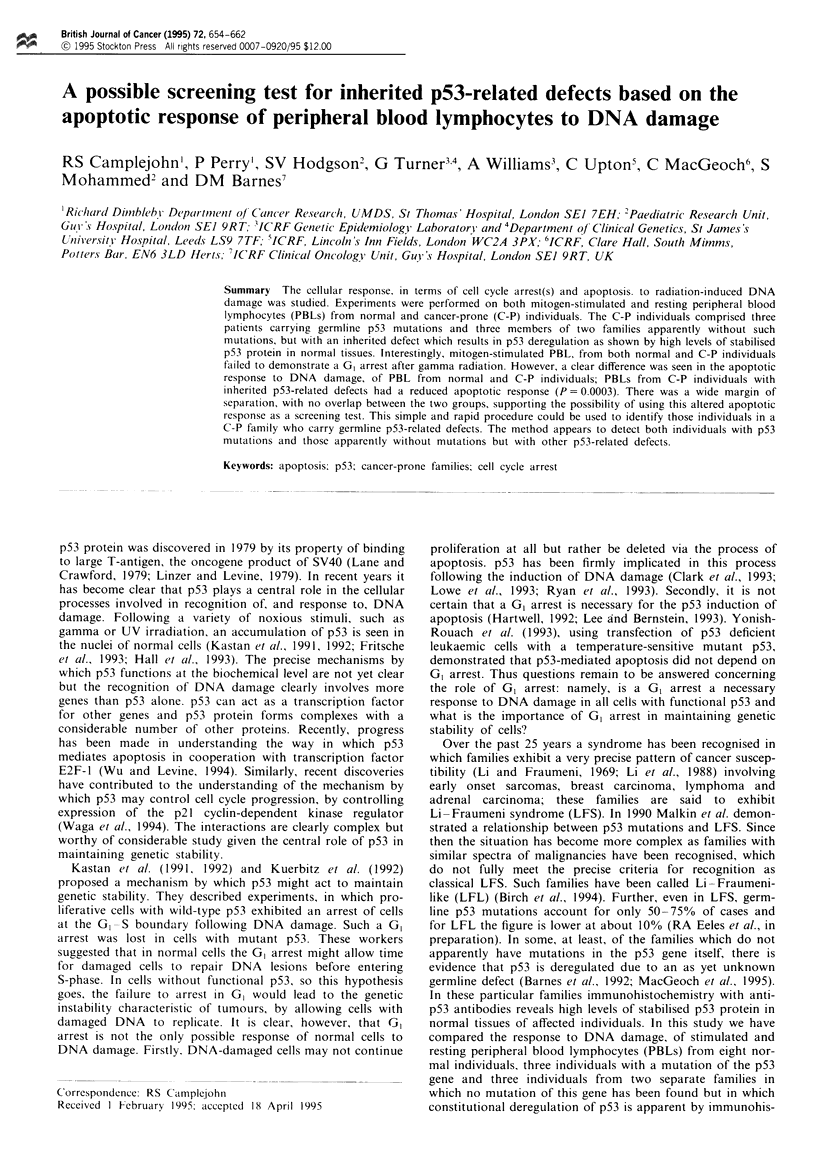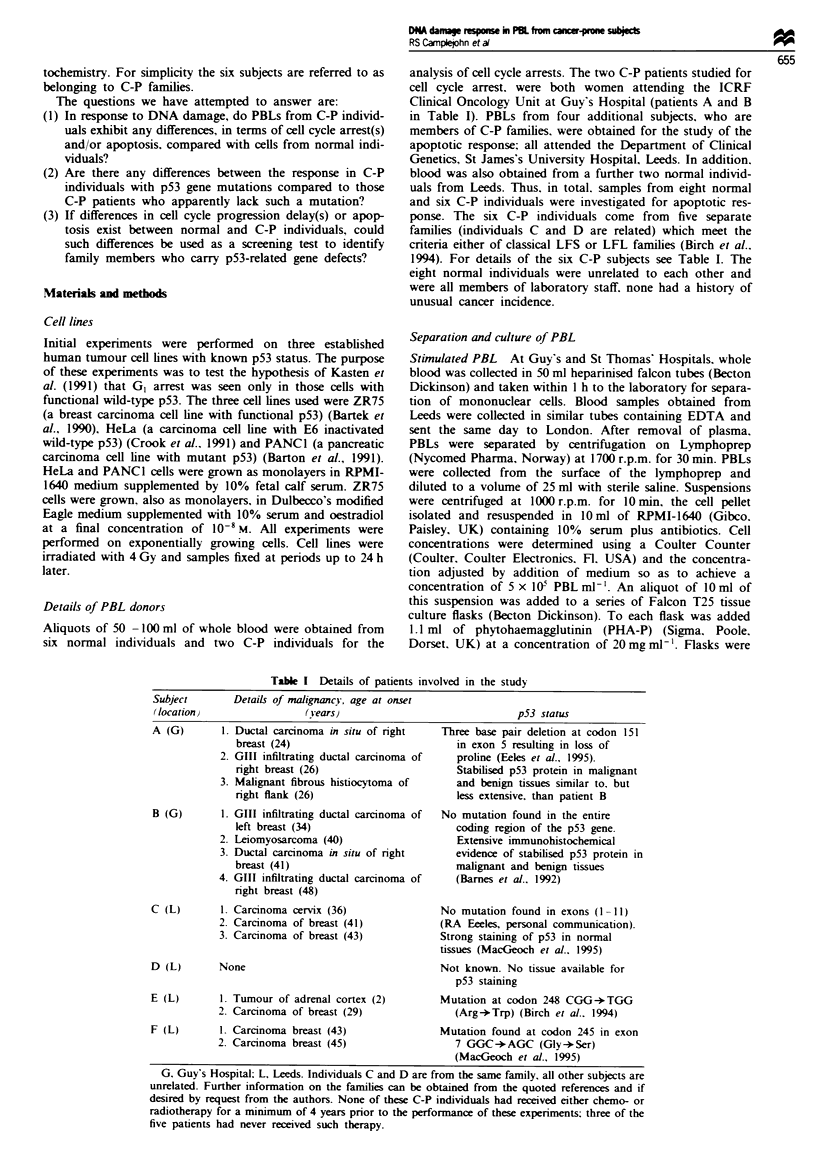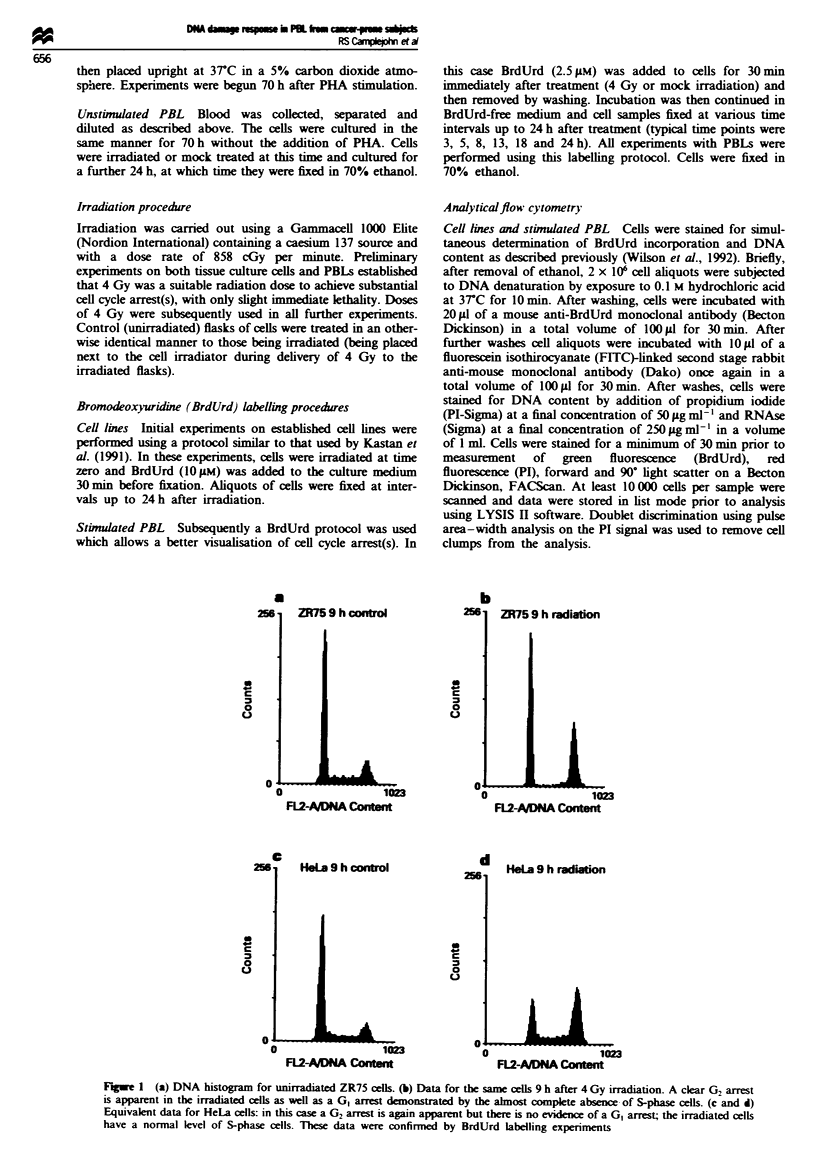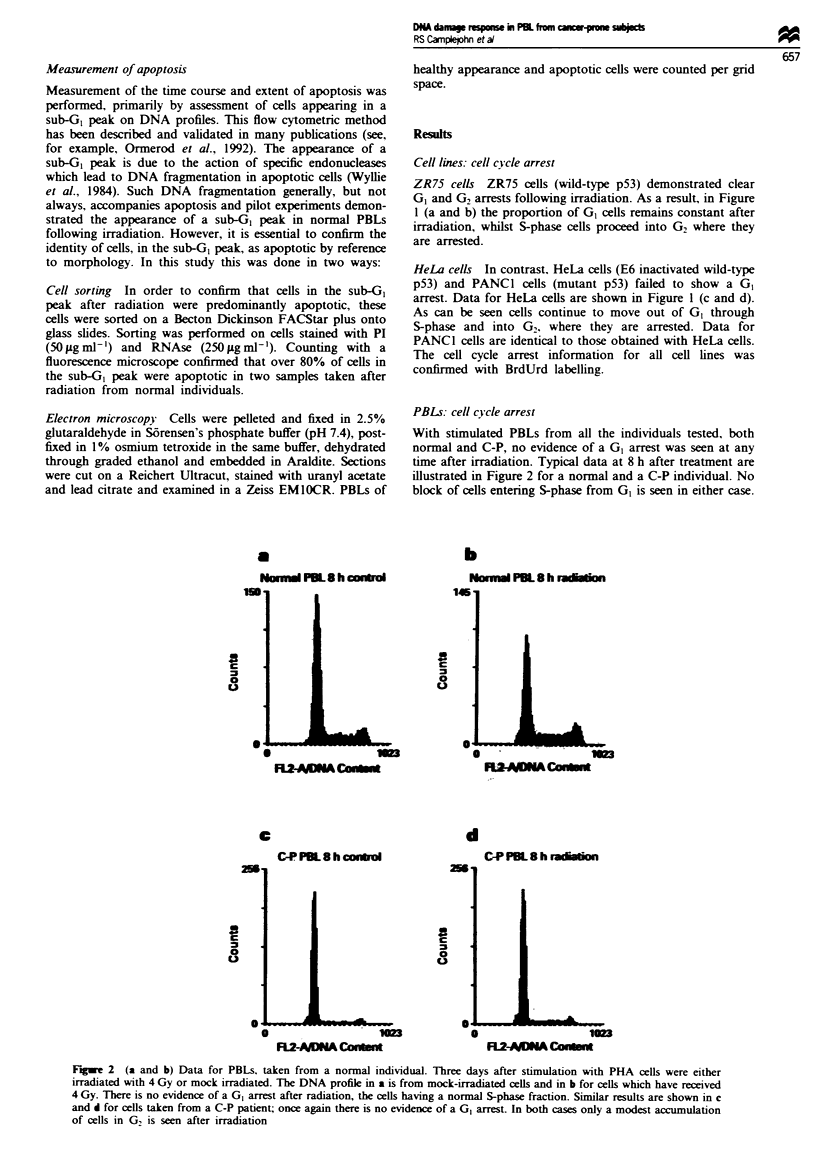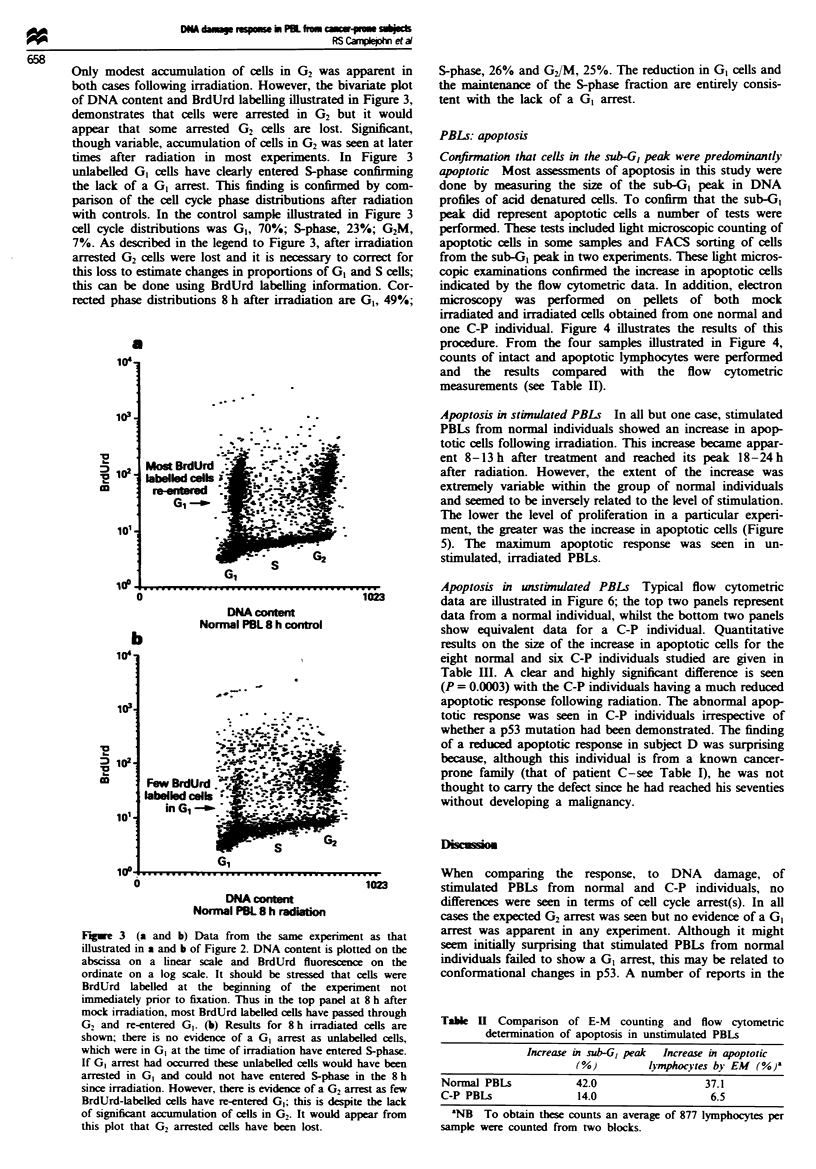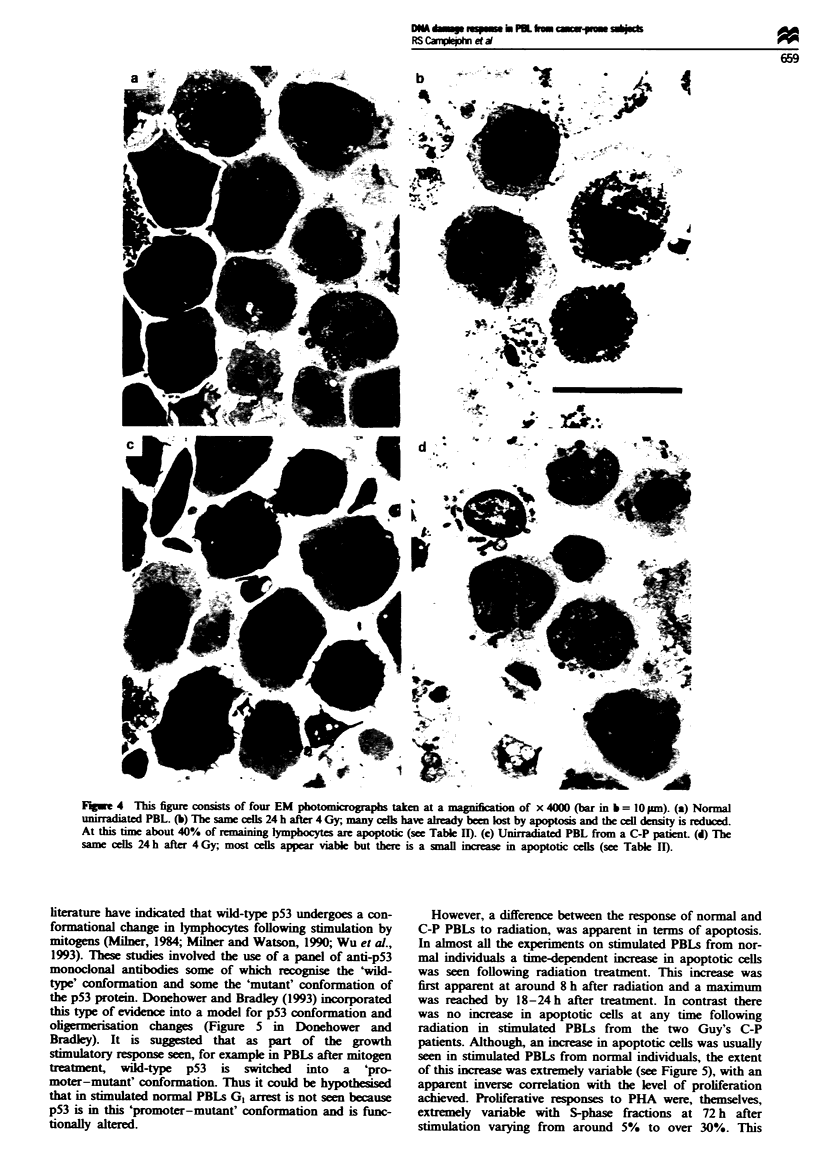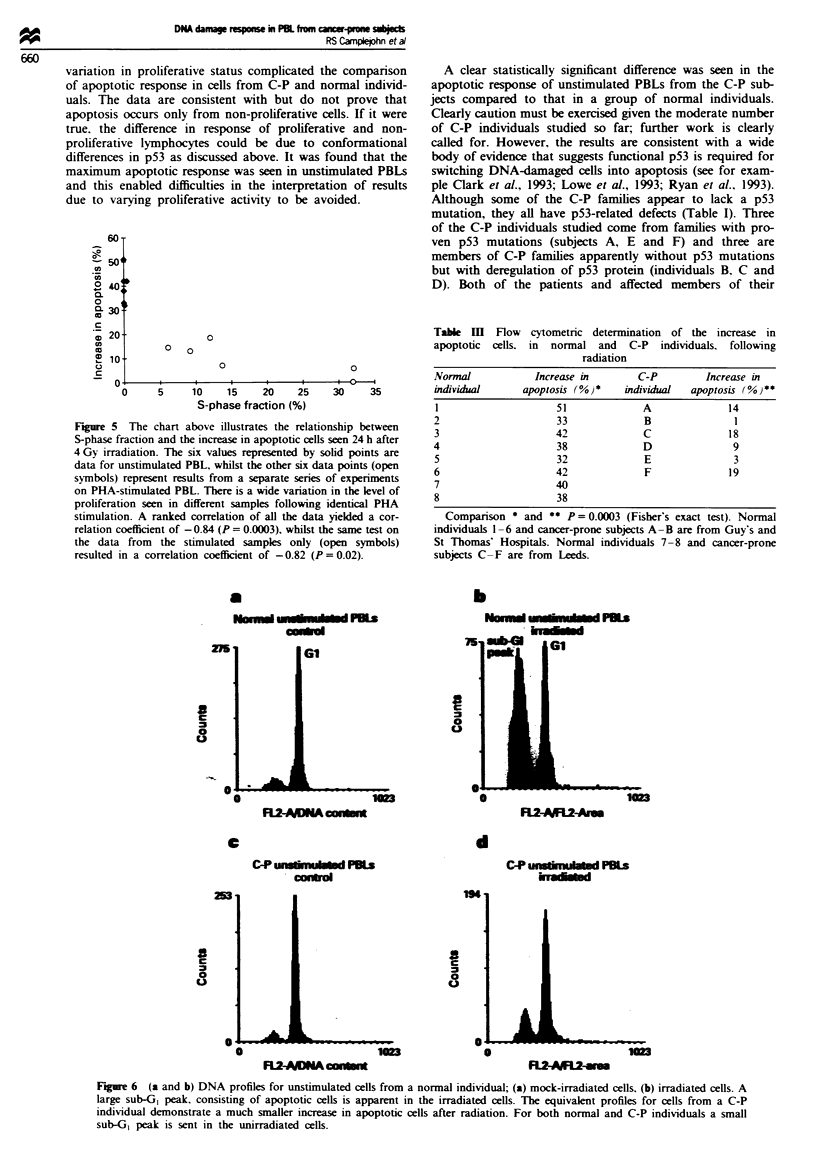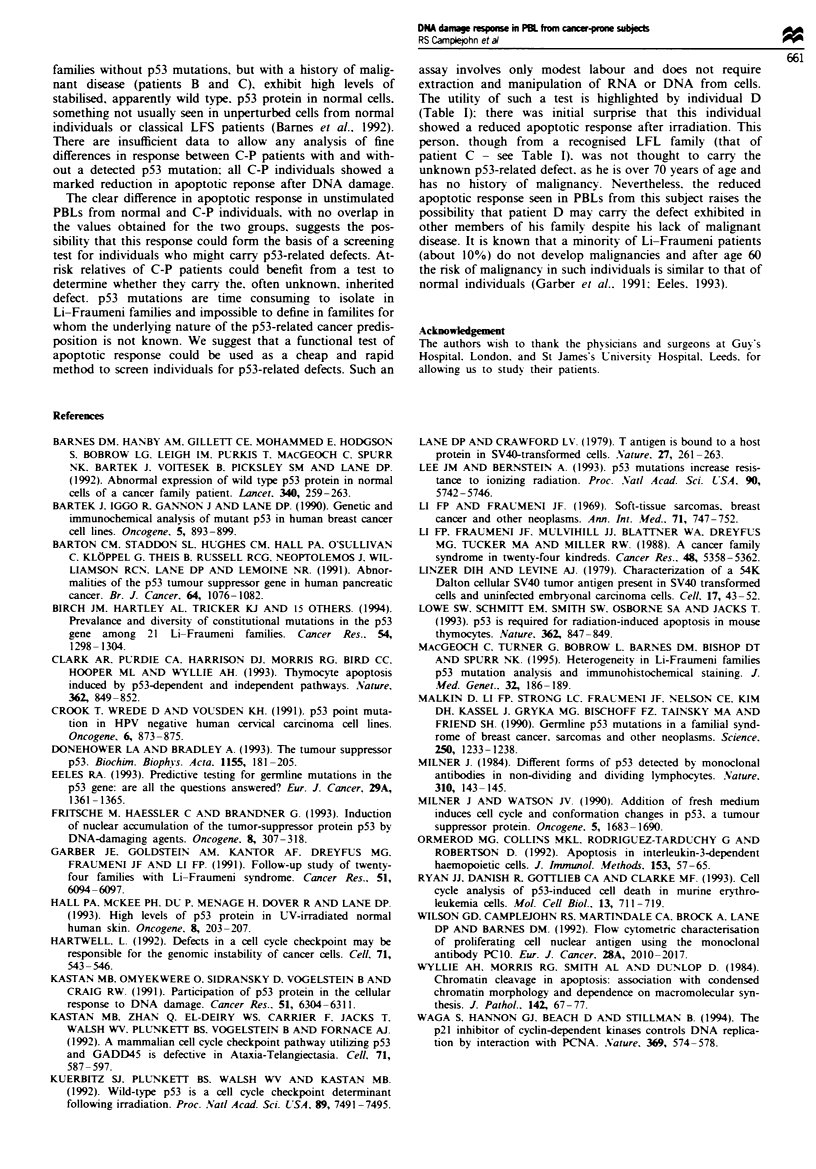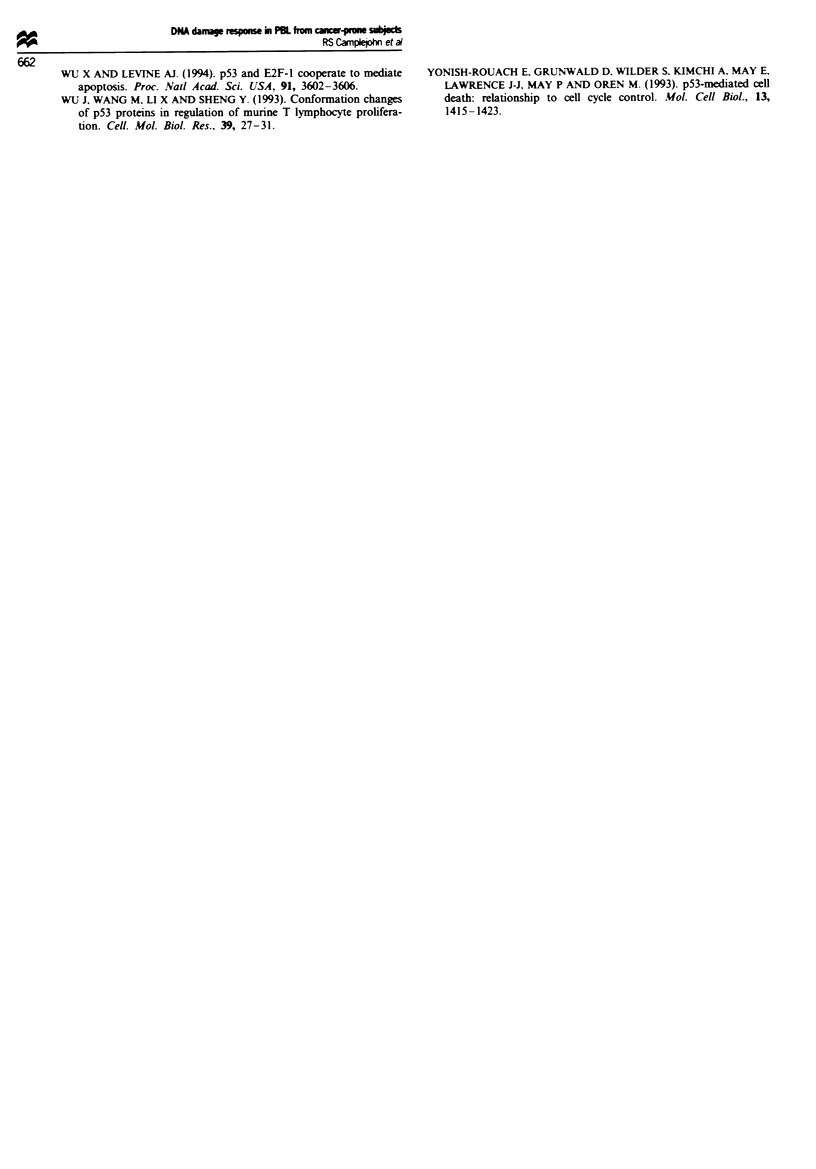# A possible screening test for inherited p53-related defects based on the apoptotic response of peripheral blood lymphocytes to DNA damage.

**DOI:** 10.1038/bjc.1995.390

**Published:** 1995-09

**Authors:** R. S. Camplejohn, P. Perry, S. V. Hodgson, G. Turner, A. Williams, C. Upton, C. MacGeoch, S. Mohammed, D. M. Barnes

**Affiliations:** Richard Dimbleby Department of Cancer Research, UMDS, St Thomas' Hospital, London, UK.

## Abstract

**Images:**


British Journal of Cancer (1995) 72, 654-662
© 1995 Stockton Press All rights reserved 0007-0920/95 $12.00

### A possible screening test for inherited p53-related defects based on the apoptotic response of peripheral blood lymphocytes to DNA damage

RS Camplejohn[1], P Perry[1], SV Hodgson[2], G Turner[3,4], A Williams[3], C Upton[5], C MacGeoch[6], S Mohammed[2] and DM Barnes[7]

[1]*Richard Dimbleby Department of Cancer Research, UMDS, St Thomas' Hospital, London SE1 7EH;* [2]*Paediatric Research Unit, Guy's Hospital, London SE1 9RT;* [3]*ICRF Genetic Epidemiology Laboratory and* [4]*Department of Clinical Genetics, St James's University Hospital, Leeds LS9 7TF;* [5]*ICRF, Lincoln's Inn Fields, London WC2A 3PX;* [6]*ICRF, Clare Hall, South Mimms, Potters Bar, EN6 3LD Herts;* [7]*ICRF Clinical Oncology Unit, Guy's Hospital, London SE1 9RT, UK*

**Summary** The cellular response, in terms of cell cycle arrest(s) and apoptosis, to radiation-induced DNA damage was studied. Experiments were performed on both mitogen-stimulated and resting peripheral blood lymphocytes (PBLs) from normal and cancer-prone (C-P) individuals. The C-P individuals comprised three patients carrying germline p53 mutations and three members of two families apparently without such mutations, but with an inherited defect which results in p53 deregulation as shown by high levels of stabilised p53 protein in normal tissues. Interestingly, mitogen-stimulated PBL, from both normal and C-P individuals failed to demonstrate a $G_1$ arrest after gamma radiation. However, a clear difference was seen in the apoptotic response to DNA damage, of PBL from normal and C-P individuals; PBLs from C-P individuals with inherited p53-related defects had a reduced apoptotic response ($P = 0.0003$). There was a wide margin of separation, with no overlap between the two groups, supporting the possibility of using this altered apoptotic response as a screening test. This simple and rapid procedure could be used to identify those individuals in a C-P family who carry germline p53-related defects. The method appears to detect both individuals with p53 mutations and those apparently without mutations but with other p53-related defects.

**Keywords:** apoptosis; p53; cancer-prone families; cell cycle arrest p53 protein was discovered in 1979 by its property of binding to large T-antigen, the oncogene product of SV40 (Lane and Crawford, 1979; Linzer and Levine, 1979). In recent years it has become clear that p53 plays a central role in the cellular processes involved in recognition of, and response to, DNA damage. Following a variety of noxious stimuli, such as gamma or UV irradiation, an accumulation of p53 is seen in the nuclei of normal cells (Kastan *et al.*, 1991, 1992; Fritsche *et al.*, 1993; Hall *et al.*, 1993). The precise mechanisms by which p53 functions at the biochemical level are not yet clear but the recognition of DNA damage clearly involves more genes than p53 alone. p53 can act as a transcription factor for other genes and p53 protein forms complexes with a considerable number of other proteins. Recently, progress has been made in understanding the way in which p53 mediates apoptosis in cooperation with transcription factor E2F-1 (Wu and Levine, 1994). Similarly, recent discoveries have contributed to the understanding of the mechanism by which p53 may control cell cycle progression, by controlling expression of the p21 cyclin-dependent kinase regulator (Waga *et al.*, 1994). The interactions are clearly complex but worthy of considerable study given the central role of p53 in maintaining genetic stability.

Kastan *et al.* (1991, 1992) and Kuerbitz *et al.* (1992) proposed a mechanism by which p53 might act to maintain genetic stability. They described experiments, in which proliferative cells with wild-type p53 exhibited an arrest of cells at the $G_1$–S boundary following DNA damage. Such a $G_1$ arrest was lost in cells with mutant p53. These workers suggested that in normal cells the $G_1$ arrest might allow time for damaged cells to repair DNA lesions before entering S-phase. In cells without functional p53, so this hypothesis goes, the failure to arrest in $G_1$ would lead to the genetic instability characteristic of tumours, by allowing cells with damaged DNA to replicate. It is clear, however, that $G_1$ arrest is not the only possible response of normal cells to DNA damage. Firstly, DNA-damaged cells may not continue proliferation at all but rather be deleted via the process of apoptosis. p53 has been firmly implicated in this process following the induction of DNA damage (Clark *et al.*, 1993; Lowe *et al.*, 1993; Ryan *et al.*, 1993). Secondly, it is not certain that a $G_1$ arrest is necessary for the p53 induction of apoptosis (Hartwell, 1992; Lee and Bernstein, 1993). Yonish-Rouach *et al.* (1993), using transfection of p53 deficient leukaemic cells with a temperature-sensitive mutant p53, demonstrated that p53-mediated apoptosis did not depend on $G_1$ arrest. Thus questions remain to be answered concerning the role of $G_1$ arrest: namely, is a $G_1$ arrest a necessary response to DNA damage in all cells with functional p53 and what is the importance of $G_1$ arrest in maintaining genetic stability of cells?

Over the past 25 years a syndrome has been recognised in which families exhibit a very precise pattern of cancer susceptibility (Li and Fraumeni, 1969; Li *et al.*, 1988) involving early onset sarcomas, breast carcinoma, lymphoma and adrenal carcinoma; these families are said to exhibit Li–Fraumeni syndrome (LFS). In 1990 Malkin *et al.* demonstrated a relationship between p53 mutations and LFS. Since then the situation has become more complex as families with similar spectra of malignancies have been recognised, which do not fully meet the precise criteria for recognition as classical LFS. Such families have been called Li–Fraumeni-like (LFL) (Birch *et al.*, 1994). Further, even in LFS, germline p53 mutations account for only 50–75% of cases and for LFL the figure is lower at about 10% (RA Eeles *et al.*, in preparation). In some, at least, of the families which do not apparently have mutations in the p53 gene itself, there is evidence that p53 is deregulated due to an as yet unknown germline defect (Barnes *et al.*, 1992; MacGeoch *et al.*, 1995). In these particular families immunohistochemistry with anti-p53 antibodies reveals high levels of stabilised p53 protein in normal tissues of affected individuals. In this study we have compared the response to DNA damage, of stimulated and resting peripheral blood lymphocytes (PBLs) from eight normal individuals, three individuals with a mutation of the p53 gene and three individuals from two separate families in which no mutation of this gene has been found but in which constitutional deregulation of p53 is apparent by immunohis-

Correspondence: RS Camplejohn

Received 1 February 1995; accepted 18 April 1995

tochemistry. For simplicity the six subjects are referred to as belonging to C-P families.

The questions we have attempted to answer are:
(1) In response to DNA damage, do PBLs from C-P individuals exhibit any differences, in terms of cell cycle arrest(s) and/or apoptosis, compared with cells from normal individuals?
(2) Are there any differences between the response in C-P individuals with p53 gene mutations compared to those C-P patients who apparently lack such a mutation?
(3) If differences in cell cycle progression delay(s) or apoptosis exist between normal and C-P individuals, could such differences be used as a screening test to identify family members who carry p53-related gene defects?

### Materials and methods

### *Cell lines*

Initial experiments were performed on three established human tumour cell lines with known p53 status. The purpose of these experiments was to test the hypothesis of Kasten *et al.* (1991) that $G_1$ arrest was seen only in those cells with functional wild-type p53. The three cell lines used were ZR75 (a breast carcinoma cell line with functional p53) (Bartek *et al.*, 1990), HeLa (a carcinoma cell line with E6 inactivated wild-type p53) (Crook *et al.*, 1991) and PANC1 (a pancreatic carcinoma cell line with mutant p53) (Barton *et al.*, 1991). HeLa and PANC1 cells were grown as monolayers in RPMI-1640 medium supplemented by 10% fetal calf serum. ZR75 cells were grown, also as monolayers, in Dulbecco's modified Eagle medium supplemented with 10% serum and oestradiol at a final concentration of $10^{-8}$ M. All experiments were performed on exponentially growing cells. Cell lines were irradiated with 4 Gy and samples fixed at periods up to 24 h later.

### *Details of PBL donors*

Aliquots of 50 – 100 ml of whole blood were obtained from six normal individuals and two C-P individuals for the analysis of cell cycle arrests. The two C-P patients studied for cell cycle arrest, were both women attending the ICRF Clinical Oncology Unit at Guy's Hospital (patients A and B in Table I). PBLs from four additional subjects, who are members of C-P families, were obtained for the study of the apoptotic response; all attended the Department of Clinical Genetics, St James's University Hospital, Leeds. In addition, blood was also obtained from a further two normal individuals from Leeds. Thus, in total, samples from eight normal and six C-P individuals were investigated for apoptotic response. The six C-P individuals come from five separate families (individuals C and D are related) which meet the criteria either of classical LFS or LFL families (Birch *et al.*, 1994). For details of the six C-P subjects see Table I. The eight normal individuals were unrelated to each other and were all members of laboratory staff, none had a history of unusual cancer incidence.

### *Separation and culture of PBL*

*Stimulated PBL* At Guy's and St Thomas' Hospitals, whole blood was collected in 50 ml heparinised falcon tubes (Becton Dickinson) and taken within 1 h to the laboratory for separation of mononuclear cells. Blood samples obtained from Leeds were collected in similar tubes containing EDTA and sent the same day to London. After removal of plasma, PBLs were separated by centrifugation on Lymphoprep (Nycomed Pharma, Norway) at 1700 r.p.m. for 30 min. PBLs were collected from the surface of the lymphoprep and diluted to a volume of 25 ml with sterile saline. Suspensions were centrifuged at 1000 r.p.m. for 10 min, the cell pellet isolated and resuspended in 10 ml of RPMI-1640 (Gibco, Paisley, UK) containing 10% serum plus antibiotics. Cell concentrations were determined using a Coulter Counter (Coulter, Coulter Electronics, Fl, USA) and the concentration adjusted by addition of medium so as to achieve a concentration of $5 \times 10^5$ PBL $ml^{-1}$. An aliquot of 10 ml of this suspension was added to a series of Falcon T25 tissue culture flasks (Becton Dickinson). To each flask was added 1.1 ml of phytohaemagglutinin (PHA-P) (Sigma, Poole, Dorset, UK) at a concentration of 20 mg $ml^{-1}$. Flasks were

**Table I** Details of patients involved in the study

| *Subject (location)* | *Details of malignancy, age at onset (years)* | *p53 status* |
|---|---|---|
| A (G) | 1. Ductal carcinoma *in situ* of right breast (24)<br>2. GIII infiltrating ductal carcinoma of right breast (26)<br>3. Malignant fibrous histiocytoma of right flank (26) | Three base pair deletion at codon 151 in exon 5 resulting in loss of proline (Eeles *et al.*, 1995). Stabilised p53 protein in malignant and benign tissues similar to, but less extensive, than patient B |
| B (G) | 1. GIII infiltrating ductal carcinoma of left breast (34)<br>2. Leiomyosarcoma (40)<br>3. Ductal carcinoma *in situ* of right breast (41)<br>4. GIII infiltrating ductal carcinoma of right breast (48) | No mutation found in the entire coding region of the p53 gene. Extensive immunohistochemical evidence of stabilised p53 protein in malignant and benign tissues (Barnes *et al.*, 1992) |
| C (L) | 1. Carcinoma cervix (36)<br>2. Carcinoma of breast (41)<br>3. Carcinoma of breast (43) | No mutation found in exons (1 – 11) (RA Eeeles, personal communication). Strong staining of p53 in normal tissues (MacGeoch *et al.*, 1995) |
| D (L) | None | Not known. No tissue available for p53 staining |
| E (L) | 1. Tumour of adrenal cortex (2)<br>2. Carcinoma of breast (29) | Mutation at codon 248 CGG→TGG (Arg→Trp) (Birch *et al.*, 1994) |
| F (L) | 1. Carcinoma breast (43)<br>2. Carcinoma breast (45) | Mutation found at codon 245 in exon 7 GGC→AGC (Gly→Ser) (MacGeoch *et al.*, 1995) |

G, Guy's Hospital; L, Leeds. Individuals C and D are from the same family, all other subjects are unrelated. Further information on the families can be obtained from the quoted references and if desired by request from the authors. None of these C-P individuals had received either chemo- or radiotherapy for a minimum of 4 years prior to the performance of these experiments; three of the five patients had never received such therapy.

then placed upright at 37°C in a 5% carbon dioxide atmosphere. Experiments were begun 70 h after PHA stimulation.

*Unstimulated PBL* Blood was collected, separated and diluted as described above. The cells were cultured in the same manner for 70 h without the addition of PHA. Cells were irradiated or mock treated at this time and cultured for a further 24 h, at which time they were fixed in 70% ethanol.

### *Irradiation procedure*

Irradiation was carried out using a Gammacell 1000 Elite (Nordion International) containing a caesium 137 source and with a dose rate of 858 cGy per minute. Preliminary experiments on both tissue culture cells and PBLs established that 4 Gy was a suitable radiation dose to achieve substantial cell cycle arrest(s), with only slight immediate lethality. Doses of 4 Gy were subsequently used in all further experiments. Control (unirradiated) flasks of cells were treated in an otherwise identical manner to those being irradiated (being placed next to the cell irradiator during delivery of 4 Gy to the irradiated flasks).

### *Bromodeoxyuridine (BrdUrd) labelling procedures*

*Cell lines* Initial experiments on established cell lines were performed using a protocol similar to that used by Kastan *et al.* (1991). In these experiments, cells were irradiated at time zero and BrdUrd (10 μM) was added to the culture medium 30 min before fixation. Aliquots of cells were fixed at intervals up to 24 h after irradiation.

*Stimulated PBL* Subsequently a BrdUrd protocol was used which allows a better visualisation of cell cycle arrest(s). In this case BrdUrd (2.5 μM) was added to cells for 30 min immediately after treatment (4 Gy or mock irradiation) and then removed by washing. Incubation was then continued in BrdUrd-free medium and cell samples fixed at various time intervals up to 24 h after treatment (typical time points were 3, 5, 8, 13, 18 and 24 h). All experiments with PBLs were performed using this labelling protocol. Cells were fixed in 70% ethanol.

### *Analytical flow cytometry*

*Cell lines and stimulated PBL* Cells were stained for simultaneous determination of BrdUrd incorporation and DNA content as described previously (Wilson *et al.*, 1992). Briefly, after removal of ethanol, 2 × $10^6$ cell aliquots were subjected to DNA denaturation by exposure to 0.1 M hydrochloric acid at 37°C for 10 min. After washing, cells were incubated with 20 μl of a mouse anti-BrdUrd monoclonal antibody (Becton Dickinson) in a total volume of 100 μl for 30 min. After further washes cell aliquots were incubated with 10 μl of a fluorescein isothirocyanate (FITC)-linked second stage rabbit anti-mouse monoclonal antibody (Dako) once again in a total volume of 100 μl for 30 min. After washes, cells were stained for DNA content by addition of propidium iodide (PI-Sigma) at a final concentration of 50 $\mu g\ ml^{-1}$ and RNAse (Sigma) at a final concentration of 250 $\mu g\ ml^{-1}$ in a volume of 1 ml. Cells were stained for a minimum of 30 min prior to measurement of green fluorescence (BrdUrd), red fluorescence (PI), forward and 90° light scatter on a Becton Dickinson, FACScan. At least 10 000 cells per sample were scanned and data were stored in list mode prior to analysis using LYSIS II software. Doublet discrimination using pulse area–width analysis on the PI signal was used to remove cell clumps from the analysis.

**Figure 1** (**a**) DNA histogram for unirradiated ZR75 cells. (**b**) Data for the same cells 9 h after 4 Gy irradiation. A clear $G_2$ arrest is apparent in the irradiated cells as well as a $G_1$ arrest demonstrated by the almost complete absence of S-phase cells. (**c** and **d**) Equivalent data for HeLa cells: in this case a $G_2$ arrest is again apparent but there is no evidence of a $G_1$ arrest; the irradiated cells have a normal level of S-phase cells. These data were confirmed by BrdUrd labelling experiments

*Measurement of apoptosis*

Measurement of the time course and extent of apoptosis was performed, primarily by assessment of cells appearing in a sub-$G_1$ peak on DNA profiles. This flow cytometric method has been described and validated in many publications (see, for example, Ormerod *et al.*, 1992). The appearance of a sub-$G_1$ peak is due to the action of specific endonucleases which lead to DNA fragmentation in apoptotic cells (Wyllie *et al.*, 1984). Such DNA fragmentation generally, but not always, accompanies apoptosis and pilot experiments demonstrated the appearance of a sub-$G_1$ peak in normal PBLs following irradiation. However, it is essential to confirm the identity of cells, in the sub-$G_1$ peak, as apoptotic by reference to morphology. In this study this was done in two ways:

*Cell sorting* In order to confirm that cells in the sub-$G_1$ peak after radiation were predominantly apoptotic, these cells were sorted on a Becton Dickinson FACStar plus onto glass slides. Sorting was performed on cells stained with PI (50 $\mu g\ ml^{-1}$) and RNAse (250 $\mu g\ ml^{-1}$). Counting with a fluorescence microscope confirmed that over 80% of cells in the sub-$G_1$ peak were apoptotic in two samples taken after radiation from normal individuals.

*Electron microscopy* Cells were pelleted and fixed in 2.5% glutaraldehyde in Sörensen's phosphate buffer (pH 7.4), post-fixed in 1% osmium tetroxide in the same buffer, dehydrated through graded ethanol and embedded in Araldite. Sections were cut on a Reichert Ultracut, stained with uranyl acetate and lead citrate and examined in a Zeiss EM10CR. PBLs of healthy appearance and apoptotic cells were counted per grid space.

### Results

*Cell lines: cell cycle arrest*

*ZR75 cells* ZR75 cells (wild-type p53) demonstrated clear $G_1$ and $G_2$ arrests following irradiation. As a result, in Figure 1 (a and b) the proportion of $G_1$ cells remains constant after irradiation, whilst S-phase cells proceed into $G_2$ where they are arrested.

*HeLa cells* In contrast, HeLa cells (E6 inactivated wild-type p53) and PANC1 cells (mutant p53) failed to show a $G_1$ arrest. Data for HeLa cells are shown in Figure 1 (c and d). As can be seen cells continue to move out of $G_1$ through S-phase and into $G_2$, where they are arrested. Data for PANC1 cells are identical to those obtained with HeLa cells. The cell cycle arrest information for all cell lines was confirmed with BrdUrd labelling.

*PBLs: cell cycle arrest*

With stimulated PBLs from all the individuals tested, both normal and C-P, no evidence of a $G_1$ arrest was seen at any time after irradiation. Typical data at 8 h after treatment are illustrated in Figure 2 for a normal and a C-P individual. No block of cells entering S-phase from $G_1$ is seen in either case.

**Figure 2** (**a** and **b**) Data for PBLs, taken from a normal individual. Three days after stimulation with PHA cells were either irradiated with 4 Gy or mock irradiated. The DNA profile in **a** is from mock-irradiated cells and in **b** for cells which have received 4 Gy. There is no evidence of a $G_1$ arrest after radiation, the cells having a normal S-phase fraction. Similar results are shown in **c** and **d** for cells taken from a C-P patient; once again there is no evidence of a $G_1$ arrest. In both cases only a modest accumulation of cells in $G_2$ is seen after irradiation

Only modest accumulation of cells in $G_2$ was apparent in both cases following irradiation. However, the bivariate plot of DNA content and BrdUrd labelling illustrated in Figure 3, demonstrates that cells were arrested in $G_2$ but it would appear that some arrested $G_2$ cells are lost. Significant, though variable, accumulation of cells in $G_2$ was seen at later times after radiation in most experiments. In Figure 3 unlabelled $G_1$ cells have clearly entered S-phase confirming the lack of a $G_1$ arrest. This finding is confirmed by comparison of the cell cycle phase distributions after radiation with controls. In the control sample illustrated in Figure 3 cell cycle distributions was $G_1$, 70%; S-phase, 23%; $G_2M$, 7%. As described in the legend to Figure 3, after irradiation arrested $G_2$ cells were lost and it is necessary to correct for this loss to estimate changes in proportions of $G_1$ and S cells; this can be done using BrdUrd labelling information. Corrected phase distributions 8 h after irradiation are $G_1$, 49%; S-phase, 26% and $G_2$/M, 25%. The reduction in $G_1$ cells and the maintenance of the S-phase fraction are entirely consistent with the lack of a $G_1$ arrest.

**Figure 3** (**a** and **b**) Data from the same experiment as that illustrated in **a** and **b** of Figure 2. DNA content is plotted on the abscissa on a linear scale and BrdUrd fluorescence on the ordinate on a log scale. It should be stressed that cells were BrdUrd labelled at the beginning of the experiment not immediately prior to fixation. Thus in the top panel at 8 h after mock irradiation, most BrdUrd labelled cells have passed through $G_2$ and re-entered $G_1$. (**b**) Results for 8 h irradiated cells are shown; there is no evidence of a $G_1$ arrest as unlabelled cells, which were in $G_1$ at the time of irradiation have entered S-phase. If $G_1$ arrest had occurred these unlabelled cells would have been arrested in $G_1$ and could not have entered S-phase in the 8 h since irradiation. However, there is evidence of a $G_2$ arrest as few BrdUrd-labelled cells have re-entered $G_1$; this is despite the lack of significant accumulation of cells in $G_2$. It would appear from this plot that $G_2$ arrested cells have been lost.

*PBLs: apoptosis*

*Confirmation that cells in the sub-$G_1$ peak were predominantly apoptotic* Most assessments of apoptosis in this study were done by measuring the size of the sub-$G_1$ peak in DNA profiles of acid denatured cells. To confirm that the sub-$G_1$ peak did represent apoptotic cells a number of tests were performed. These tests included light microscopic counting of apoptotic cells in some samples and FACS sorting of cells from the sub-$G_1$ peak in two experiments. These light microscopic examinations confirmed the increase in apoptotic cells indicated by the flow cytometric data. In addition, electron microscopy was performed on pellets of both mock irradiated and irradiated cells obtained from one normal and one C-P individual. Figure 4 illustrates the results of this procedure. From the four samples illustrated in Figure 4, counts of intact and apoptotic lymphocytes were performed and the results compared with the flow cytometric measurements (see Table II).

*Apoptosis in stimulated PBLs* In all but one case, stimulated PBLs from normal individuals showed an increase in apoptotic cells following irradiation. This increase became apparent 8–13 h after treatment and reached its peak 18–24 h after radiation. However, the extent of the increase was extremely variable within the group of normal individuals and seemed to be inversely related to the level of stimulation. The lower the level of proliferation in a particular experiment, the greater was the increase in apoptotic cells (Figure 5). The maximum apoptotic response was seen in unstimulated, irradiated PBLs.

*Apoptosis in unstimulated PBLs* Typical flow cytometric data are illustrated in Figure 6; the top two panels represent data from a normal individual, whilst the bottom two panels show equivalent data for a C-P individual. Quantitative results on the size of the increase in apoptotic cells for the eight normal and six C-P individuals studied are given in Table III. A clear and highly significant difference is seen ($P = 0.0003$) with the C-P individuals having a much reduced apoptotic response following radiation. The abnormal apoptotic response was seen in C-P individuals irrespective of whether a p53 mutation had been demonstrated. The finding of a reduced apoptotic response in subject D was surprising because, although this individual is from a known cancer-prone family (that of patient C–see Table I), he was not thought to carry the defect since he had reached his seventies without developing a malignancy.

### Discussion

When comparing the response, to DNA damage, of stimulated PBLs from normal and C-P individuals, no differences were seen in terms of cell cycle arrest(s). In all cases the expected $G_2$ arrest was seen but no evidence of a $G_1$ arrest was apparent in any experiment. Although it might seem initially surprising that stimulated PBLs from normal individuals failed to show a $G_1$ arrest, this may be related to conformational changes in p53. A number of reports in the

**Table II** Comparison of E-M counting and flow cytometric determination of apoptosis in unstimulated PBLs

| | *Increase in sub-$G_1$ peak (%)* | *Increase in apoptotic lymphocytes by EM (%)*[a] |
|---|---|---|
| Normal PBLs | 42.0 | 37.1 |
| C-P PBLs | 14.0 | 6.5 |

[a]NB To obtain these counts an average of 877 lymphocytes per sample were counted from two blocks.

Figure 4 This figure consists of four EM photomicrographs taken at a magnification of × 4000 (bar in b = 10 μm). (a) Normal unirradiated PBL. (b) The same cells 24 h after 4 Gy; many cells have already been lost by apoptosis and the cell density is reduced. At this time about 40% of remaining lymphocytes are apoptotic (see Table II). (c) Unirradiated PBL from a C-P patient. (d) The same cells 24 h after 4 Gy; most cells appear viable but there is a small increase in apoptotic cells (see Table II).

literature have indicated that wild-type p53 undergoes a conformational change in lymphocytes following stimulation by mitogens (Milner, 1984; Milner and Watson, 1990; Wu *et al.*, 1993). These studies involved the use of a panel of anti-p53 monoclonal antibodies some of which recognise the 'wild-type' conformation and some the 'mutant' conformation of the p53 protein. Donehower and Bradley (1993) incorporated this type of evidence into a model for p53 conformation and oligermerisation changes (Figure 5 in Donehower and Bradley). It is suggested that as part of the growth stimulatory response seen, for example in PBLs after mitogen treatment, wild-type p53 is switched into a 'promoter–mutant' conformation. Thus it could be hypothesised that in stimulated normal PBLs $G_1$ arrest is not seen because p53 is in this 'promoter–mutant' conformation and is functionally altered.

However, a difference between the response of normal and C-P PBLs to radiation, was apparent in terms of apoptosis. In almost all the experiments on stimulated PBLs from normal individuals a time-dependent increase in apoptotic cells was seen following radiation treatment. This increase was first apparent at around 8 h after radiation and a maximum was reached by 18–24 h after treatment. In contrast there was no increase in apoptotic cells at any time following radiation in stimulated PBLs from the two Guy's C-P patients. Although, an increase in apoptotic cells was usually seen in stimulated PBLs from normal individuals, the extent of this increase was extremely variable (see Figure 5), with an apparent inverse correlation with the level of proliferation achieved. Proliferative responses to PHA were, themselves, extremely variable with S-phase fractions at 72 h after stimulation varying from around 5% to over 30%. This variation in proliferative status complicated the comparison of apoptotic response in cells from C-P and normal individuals. The data are consistent with but do not prove that apoptosis occurs only from non-proliferative cells. If it were true, the difference in response of proliferative and non-proliferative lymphocytes could be due to conformational differences in p53 as discussed above. It was found that the maximum apoptotic response was seen in unstimulated PBLs and this enabled difficulties in the interpretation of results due to varying proliferative activity to be avoided.

**Figure 5** The chart above illustrates the relationship between S-phase fraction and the increase in apoptotic cells seen 24 h after 4 Gy irradiation. The six values represented by solid points are data for unstimulated PBL, whilst the other six data points (open symbols) represent results from a separate series of experiments on PHA-stimulated PBL. There is a wide variation in the level of proliferation seen in different samples following identical PHA stimulation. A ranked correlation of all the data yielded a correlation coefficient of $-0.84$ ($P = 0.0003$), whilst the same test on the data from the stimulated samples only (open symbols) resulted in a correlation coefficient of $-0.82$ ($P = 0.02$).

A clear statistically significant difference was seen in the apoptotic response of unstimulated PBLs from the C-P subjects compared to that in a group of normal individuals. Clearly caution must be exercised given the moderate number of C-P individuals studied so far; further work is clearly called for. However, the results are consistent with a wide body of evidence that suggests functional p53 is required for switching DNA-damaged cells into apoptosis (see for example Clark *et al*., 1993; Lowe *et al*., 1993; Ryan *et al*., 1993). Although some of the C-P families appear to lack a p53 mutation, they all have p53-related defects (Table I). Three of the C-P individuals studied come from families with proven p53 mutations (subjects A, E and F) and three are members of C-P families apparently without p53 mutations but with deregulation of p53 protein (individuals B, C and D). Both of the patients and affected members of their

**Table III** Flow cytometric determination of the increase in apoptotic cells, in normal and C-P individuals, following radiation

| *Normal individual* | *Increase in apoptosis (%)** | *C-P individual* | *Increase in apoptosis (%)*** |
|---|---|---|---|
| 1 | 51 | A | 14 |
| 2 | 33 | B | 1 |
| 3 | 42 | C | 18 |
| 4 | 38 | D | 9 |
| 5 | 32 | E | 3 |
| 6 | 42 | F | 19 |
| 7 | 40 | | |
| 8 | 38 | | |

Comparison * and ** $P = 0.0003$ (Fisher's exact test). Normal individuals 1–6 and cancer-prone subjects A–B are from Guy's and St Thomas' Hospitals. Normal individuals 7–8 and cancer-prone subjects C–F are from Leeds.

**Figure 6** (**a** and **b**) DNA profiles for unstimulated cells from a normal individual; (**a**) mock-irradiated cells, (**b**) irradiated cells. A large sub-$G_1$ peak, consisting of apoptotic cells is apparent in the irradiated cells. The equivalent profiles for cells from a C-P individual demonstrate a much smaller increase in apoptotic cells after radiation. For both normal and C-P individuals a small sub-$G_1$ peak is sent in the unirradiated cells.

families without p53 mutations, but with a history of malignant disease (patients B and C), exhibit high levels of stabilised, apparently wild type, p53 protein in normal cells, something not usually seen in unperturbed cells from normal individuals or classical LFS patients (Barnes *et al.*, 1992). There are insufficient data to allow any analysis of fine differences in response between C-P patients with and without a detected p53 mutation; all C-P individuals showed a marked reduction in apoptotic reponse after DNA damage.

The clear difference in apoptotic response in unstimulated PBLs from normal and C-P individuals, with no overlap in the values obtained for the two groups, suggests the possibility that this response could form the basis of a screening test for individuals who might carry p53-related defects. At-risk relatives of C-P patients could benefit from a test to determine whether they carry the, often unknown, inherited defect. p53 mutations are time consuming to isolate in Li–Fraumeni families and impossible to define in familites for whom the underlying nature of the p53-related cancer predisposition is not known. We suggest that a functional test of apoptotic response could be used as a cheap and rapid method to screen individuals for p53-related defects. Such an assay involves only modest labour and does not require extraction and manipulation of RNA or DNA from cells. The utility of such a test is highlighted by individual D (Table I); there was initial surprise that this individual showed a reduced apoptotic response after irradiation. This person, though from a recognised LFL family (that of patient C – see Table I), was not thought to carry the unknown p53-related defect, as he is over 70 years of age and has no history of malignancy. Nevertheless, the reduced apoptotic response seen in PBLs from this subject raises the possibility that patient D may carry the defect exhibited in other members of his family despite his lack of malignant disease. It is known that a minority of Li–Fraumeni patients (about 10%) do not develop malignancies and after age 60 the risk of malignancy in such individuals is similar to that of normal individuals (Garber *et al.*, 1991; Eeles, 1993).

**Acknowledgement**

The authors wish to thank the physicians and surgeons at Guy's Hospital, London, and St James's University Hospital, Leeds, for allowing us to study their patients.

**References**

BARNES DM, HANBY AM, GILLETT CE, MOHAMMED E, HODGSON S, BOBROW LG, LEIGH IM, PURKIS T, MACGEOCH C, SPURR NK, BARTEK J, VOITESEK B, PICKSLEY SM AND LANE DP. (1992). Abnormal expression of wild type p53 protein in normal cells of a cancer family patient. *Lancet*, **340**, 259–263.

BARTEK J, IGGO R, GANNON J AND LANE DP. (1990). Genetic and immunochemical analysis of mutant p53 in human breast cancer cell lines. *Oncogene*, **5**, 893–899.

BARTON CM, STADDON SL, HUGHES CM, HALL PA, O'SULLIVAN C, KLÖPPEL G, THEIS B, RUSSELL RCG, NEOPTOLEMOS J, WILLIAMSON RCN, LANE DP AND LEMOINE NR. (1991). Abnormalities of the p53 tumour suppressor gene in human pancreatic cancer. *Br. J. Cancer*, **64**, 1076–1082.

BIRCH JM, HARTLEY AL, TRICKER KJ AND 15 OTHERS. (1994). Prevalance and diversity of constitutional mutations in the p53 gene among 21 Li–Fraumeni families. *Cancer Res.*, **54**, 1298–1304.

CLARK AR, PURDIE CA, HARRISON DJ, MORRIS RG, BIRD CC, HOOPER ML AND WYLLIE AH. (1993). Thymocyte apoptosis induced by p53-dependent and independent pathways. *Nature*, **362**, 849–852.

CROOK T, WREDE D AND VOUSDEN KH. (1991). p53 point mutation in HPV negative human cervical carcinoma cell lines. *Oncogene*, **6**, 873–875.

DONEHOWER LA AND BRADLEY A. (1993). The tumour suppressor p53. *Biochim. Biophys. Acta*, **1155**, 181–205.

EELES RA. (1993). Predictive testing for germline mutations in the p53 gene: are all the questions answered? *Eur. J. Cancer*, **29A**, 1361–1365.

FRITSCHE M, HAESSLER C AND BRANDNER G. (1993). Induction of nuclear accumulation of the tumor-suppressor protein p53 by DNA-damaging agents. *Oncogene*, **8**, 307–318.

GARBER JE, GOLDSTEIN AM, KANTOR AF, DREYFUS MG, FRAUMENI JF AND LI FP. (1991). Follow-up study of twenty-four families with Li–Fraumeni syndrome. *Cancer Res.*, **51**, 6094–6097.

HALL PA, MCKEE PH, DU P, MENAGE H, DOVER R AND LANE DP. (1993). High levels of p53 protein in UV-irradiated normal human skin. *Oncogene*, **8**, 203–207.

HARTWELL, L. (1992). Defects in a cell cycle checkpoint may be responsible for the genomic instability of cancer cells. *Cell*, **71**, 543–546.

KASTAN MB, OMYEKWERE O, SIDRANSKY D, VOGELSTEIN B AND CRAIG RW. (1991). Participation of p53 protein in the cellular response to DNA damage. *Cancer Res.*, **51**, 6304–6311.

KASTAN MB, ZHAN Q, EL-DEIRY WS, CARRIER F, JACKS T, WALSH WV, PLUNKETT BS, VOGELSTEIN B AND FORNACE AJ. (1992). A mammalian cell cycle checkpoint pathway utilizing p53 and GADD45 is defective in Ataxia-Telangiectasia. *Cell*, **71**, 587–597.

KUERBITZ SJ, PLUNKETT BS, WALSH WV AND KASTAN MB. (1992). Wild-type p53 is a cell cycle checkpoint determinant following irradiation. *Proc. Natl Acad. Sci. USA*, **89**, 7491–7495.

LANE DP AND CRAWFORD LV. (1979). T antigen is bound to a host protein in SV40-transformed cells. *Nature*, **27**, 261–263.

LEE JM AND BERNSTEIN A. (1993). p53 mutations increase resistance to ionizing radiation. *Proc. Natl Acad. Sci. USA*, **90**, 5742–5746.

LI FP AND FRAUMENI JF. (1969). Soft-tissue sarcomas, breast cancer and other neoplasms. *Ann. Int. Med.*, **71**, 747–752.

LI FP, FRAUMENI JF, MULVIHILL JJ, BLATTNER WA, DREYFUS MG, TUCKER MA AND MILLER RW. (1988). A cancer family syndrome in twenty-four kindreds. *Cancer Res.*, **48**, 5358–5362.

LINZER DIH AND LEVINE AJ. (1979). Characterization of a 54K Dalton cellular SV40 tumor antigen present in SV40 transformed cells and uninfected embryonal carcinoma cells. *Cell*, **17**, 43–52.

LOWE SW, SCHMITT EM, SMITH SW, OSBORNE SA AND JACKS T. (1993). p53 is required for radiation-induced apoptosis in mouse thymocytes. *Nature*, **362**, 847–849.

MACGEOCH C, TURNER G, BOBROW L, BARNES DM, BISHOP DT AND SPURR NK. (1995). Heterogeneity in Li-Fraumeni families p53 mutation analysis and immunohistochemical staining. *J. Med. Genet.*, **32**, 186–189.

MALKIN D, LI FP, STRONG LC, FRAUMENI JF, NELSON CE, KIM DH, KASSEL J, GRYKA MG, BISCHOFF FZ, TAINSKY MA AND FRIEND SH. (1990). Germline p53 mutations in a familial syndrome of breast cancer, sarcomas and other neoplasms. *Science*, **250**, 1233–1238.

MILNER J. (1984). Different forms of p53 detected by monoclonal antibodies in non-dividing and dividing lymphocytes. *Nature*, **310**, 143–145.

MILNER J AND WATSON JV. (1990). Addition of fresh medium induces cell cycle and conformation changes in p53, a tumour suppressor protein. *Oncogene*, **5**, 1683–1690.

ORMEROD MG, COLLINS MKL, RODRIGUEZ-TARDUCHY G AND ROBERTSON D. (1992). Apoptosis in interleukin-3-dependent haemopoietic cells. *J. Immunol. Methods*, **153**, 57–65.

RYAN JJ, DANISH R, GOTTLIEB CA AND CLARKE MF. (1993). Cell cycle analysis of p53-induced cell death in murine erythroleukemia cells. *Mol. Cell Biol.*, **13**, 711–719.

WILSON GD, CAMPLEJOHN RS, MARTINDALE CA, BROCK A, LANE DP AND BARNES DM. (1992). Flow cytometric characterisation of proliferating cell nuclear antigen using the monoclonal antibody PC10. *Eur. J. Cancer*, **28A**, 2010–2017.

WYLLIE AH, MORRIS RG, SMITH AL AND DUNLOP D. (1984). Chromatin cleavage in apoptosis: association with condensed chromatin morphology and dependence on macromolecular synthesis. *J. Pathol.*, **142**, 67–77.

WAGA S, HANNON GJ, BEACH D AND STILLMAN B. (1994). The p21 inhibitor of cyclin-dependent kinases controls DNA replication by interaction with PCNA. *Nature*, **369**, 574–578.

WU X AND LEVINE AJ. (1994). p53 and E2F-1 cooperate to mediate apoptosis. *Proc. Natl Acad. Sci. USA*, **91**, 3602–3606.
WU J, WANG M, LI X AND SHENG Y. (1993). Conformation changes of p53 proteins in regulation of murine T lymphocyte proliferation. *Cell. Mol. Biol. Res.*, **39**, 27–31.
YONISH-ROUACH E, GRUNWALD D, WILDER S, KIMCHI A, MAY E, LAWRENCE J-J, MAY P AND OREN M. (1993). p53-mediated cell death: relationship to cell cycle control. *Mol. Cell Biol.*, **13**, 1415–1423.